# Body mass index and cancer risk in patients with type 2 diabetes: a dose–response meta-analysis of cohort studies

**DOI:** 10.1038/s41598-021-81671-0

**Published:** 2021-01-28

**Authors:** Sepideh Soltani, Shima Abdollahi, Dagfinn Aune, Ahmad Jayedi

**Affiliations:** 1 Yazd Cardiovascular Research Center, Shahid Sadoughi University of Medical Sciences, Yazd, Iran; 2 Department of Nutrition and Public Health, School of Public Health, North Khorasan University of Medical Sciences, Bojnurd, Iran; 3 Department of Epidemiology and Biostatistics, Imperial College London, London, UK; 4 Department of Nutrition, Bjørknes University College, Oslo, Norway; 5 Department of Endocrinology, Morbid Obesity and Preventive Medicine, Oslo University Hospital, Ullevål, Oslo, Norway; 6 Food Safety Research Center (Salt), Semnan University of Medical Sciences, Semnan, Iran

**Keywords:** Cancer, Breast cancer, Cancer epidemiology

## Abstract

Although obesity has been associated with an increased cancer risk in the general population, the association in patients with type 2 diabetes (T2D) remains controversial. We conducted a dose–response meta-analysis of cohort studies of body mass index (BMI) and the risk of total and site-specific cancers in patients with T2D. A systematic literature search was conducted in PubMed, Scopus, and Medline until September 2020 for cohort studies on the association between BMI and cancer risk in patients with T2D. Summary relative risks (RRs) and 95% confidence intervals (CIs) were calculated using random effects models. Ten prospective and three retrospective cohort studies (3,345,031 participants and 37,412 cases) were included in the meta-analysis. Each 5-unit increase in BMI (kg/m^2^) was associated with a 6% higher risk of total cancer (RR: 1.06, 95% CI 1.01, 1.10; I^2^ = 55.4%, *n* = 6), and with a 12% increased risk in the analysis of breast cancer (RR: 1.12, 95% CI 1.05, 1.20; I^2^ = 0%, *n* = 3). The pooled RRs showed no association with prostate cancer (RR: 1.02, 95% CI 0.92, 1.13; I^2^ = 64.6%, *n* = 4), pancreatic cancer (RR: 0.97, 95% CI 0.84, 1.11; I^2^ = 71%, *n* = 3), and colorectal cancer (RR: 1.05, 95% CI 0.98, 1.13; I^2^ = 65.9%, *n* = 2). There was no indication of nonlinearity for total cancer (P_non-linearity_ = 0.99), however, there was evidence of a nonlinear association between BMI and breast cancer (P_non-linearity_ = 0.004) with steeper increases in risk from a BMI around 35 and above respectively. Higher BMI was associated with a higher risk of total, and breast cancer but not with risk of other cancers, in patients with T2D, however, further studies are needed before firm conclusions can be drawn.

## Introduction

The prevalence of high body mass index (BMI, weight in kg/height in m^2^) and diabetes has increased substantially over the past decades worldwide^1,2^. Both adiposity and diabetes are major risk factors for cancer and a range of other complications and are associated with a substantial public health burden globally^3–7^. According to the World Cancer Research Fund (WCRF) and evidence from some meta-analyses adiposity is an established risk factor for 12 different types of cancer including cancers of the oesophagus (adenocarcinoma), stomach (cardia), colorectal, gallbladder, pancreas, kidney, liver, endometrial, breast (postmenopausal), ovaries, and thyroid^8–15^. In addition, there is some evidence to suggest increased risk of leukaemia, Hodgkin's disease, non-Hodgkin's lymphoma and multiple myeloma^16^.

Type 2 diabetes is strongly related to excess body weight^17^, and has been associated with increased risk of cancers of the colorectum, pancreas, liver, gallbladder, breast and endometrium, independent of BMI^18^. Hyperinsulinemia^19^, increased circulating levels of insulin growth factor-I (IGF-I)^20^, and alterations in sex hormones and sex hormone-binding proteins^21^ are some of the proposed mechanisms through which obesity and type 2 diabetes increase the risk of cancers. Moreover, higher circulating levels of adipo-cytokines^22,23^ and inflammatory markers^24^ are the two common abnormalities in obesity and diabetes, both of which are associated with cancer development^23,24^. It has been estimated that 5.6% of all incident cancer cases were attributable to the synergistic effects of high BMI and type 2 diabetes in 2012^25^.

Although the associations between BMI and the risk of total and site-specific cancers in the general population have been widely investigated^3,9–12,15,26^, such associations in patients with existing type 2 diabetes have been less investigated and results are less clear than in general population-based studies^27–32^. Some studies have found a positive association with risk of total cancer^31–33^, however, not all studies have been consistent^29,30,34,35^, and results have been less convincing for hormone-related^27–30,32,36–38^ and colorectal cancer^29,30,32^.

To our knowledge, no systematic review and meta-analysis has assessed the association of BMI with the risk of total and site-specific cancers in patients with T2D. Thus, we aimed to clarify the strength and shape of the associations between BMI and the risk of overall and site-specific cancers in patients with T2D by systematic review and meta-analysis of cohort studies.

## Results

The initial database search yeilded 11,070 articles, of these, 55 were selected for full-text checking after removal of duplicate articles and the abstract/title screening. The Cohen’s κ coefficient was 0.75 at the abstract screening stage and 1.0 at the reviewing of the full-text.

**Figure 1:**
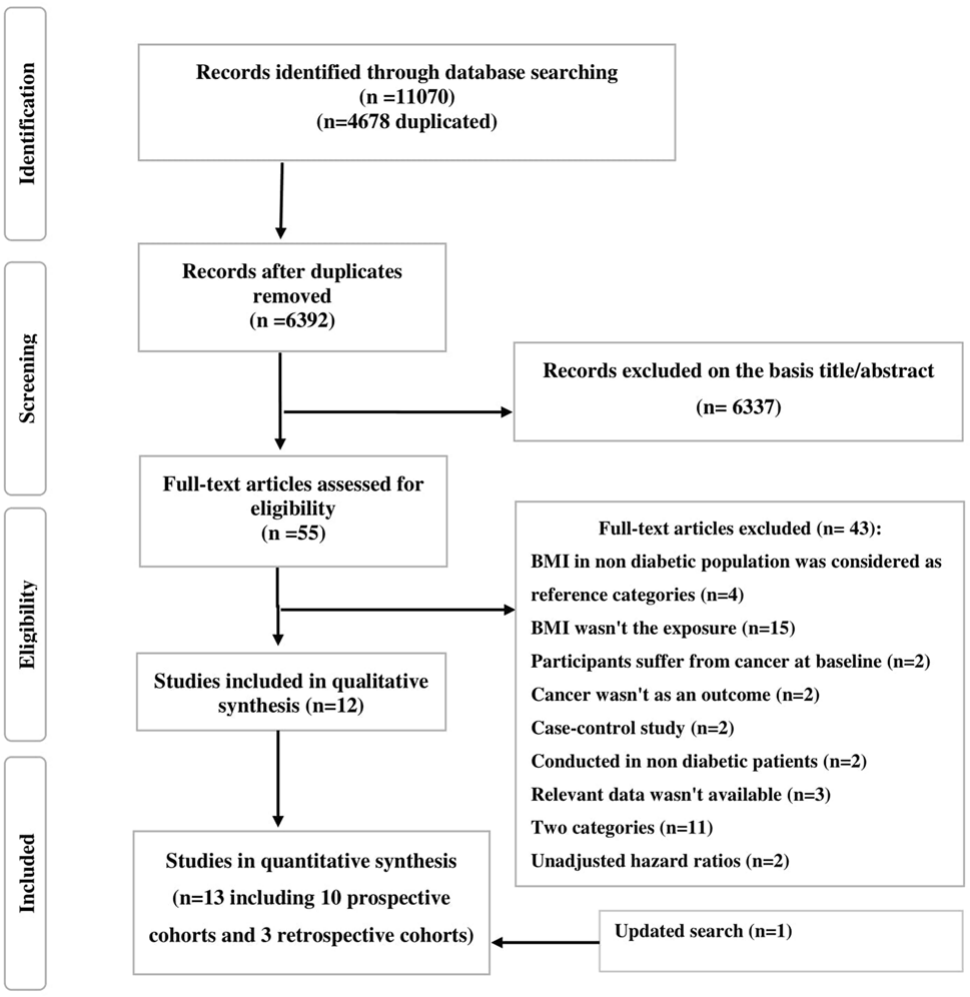
Flow diagram of the study selection process.

The study selection process and the reasons for excluding studies are given as a flowchart in Fig. 1. Exclusion reasons are provided in Supplementary Table S1. Thirteen articles^28–30,32–41^, including 3,345,031 patients with T2D with age ranging from 18 to 90 year fulfilled our inclusion criteria. Detailed characteristics of the studies included in the present meta-analysis are shown in Supplementary Table S2. Ten studies were prospective cohorts^28,32–37,39–41^ and three were retrospective cohort studies^29,30,38^ and in all of the them, measurement of BMI was performed at baseline of the study and prior to diagnosis of cancer. Six studies had an average follow-up duration of ≥ 10 years^28,33,34,37,39,40^. Four studies were conducted in North America^28,33,37,40^, four studies in Europe^32,34,36,38^, and five in Asia^29,30,35,39,41^. Two studies included only females^33,37^, three studies included only males^28,36,39^, and the remaining studies included both males and females^29,30,32,34,35,38,40,41^.The included studies were published between 2001 and 2019 and included over 37,412 cancer cases (7553 total cancer cases, 666 pancreatic cancer cases, 3387 prostate cancer cases, 839 breast cancer cases, and 24,827 colorectal cancer cases). In 10 studies, BMI was based on measured height and weight^28,30,32–39^, another three studies used self-reported anthropometry^29,40,41^. Cancer cases were recognized through national cancer registries in all studies^28–30,32–40^. Overall, eight primary studies were of high quality^28,32–34,36–39^, with a Newcastle Ottawa study quality score ranging from 7 to 9, and another four studies had moderate quality scores, with a score of five or six (Supplementary Table S3).

**Figure 2:**
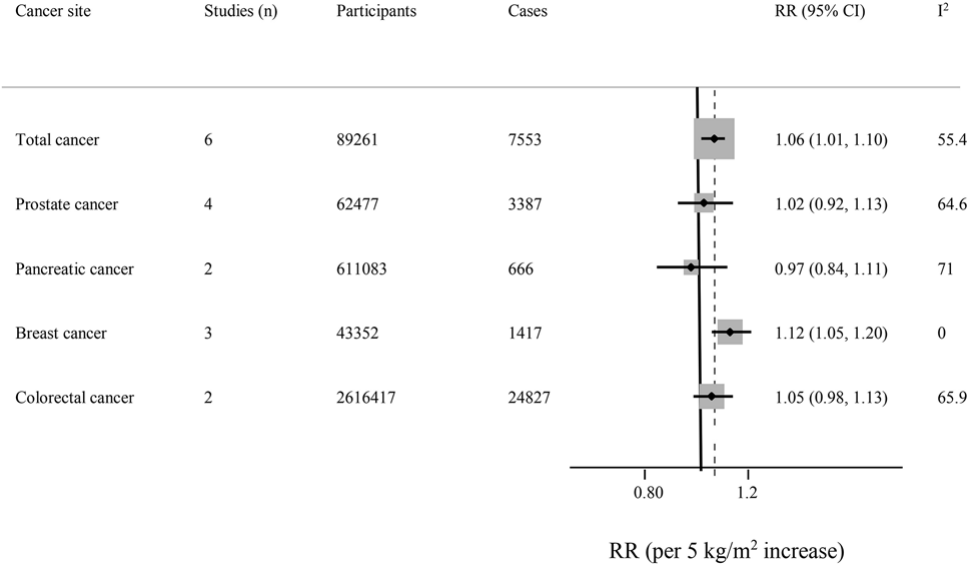
Summary risk estimates of overall and different cancer sites for each 5-unit increase in body mass index in patients with T2D.

Figure 2 show the results of summary risk estimates for total and specific cancers per 5 -unit increase in BMI (kg/m^2^) in patients with T2D.

### Total cancer

**Figure 3:**
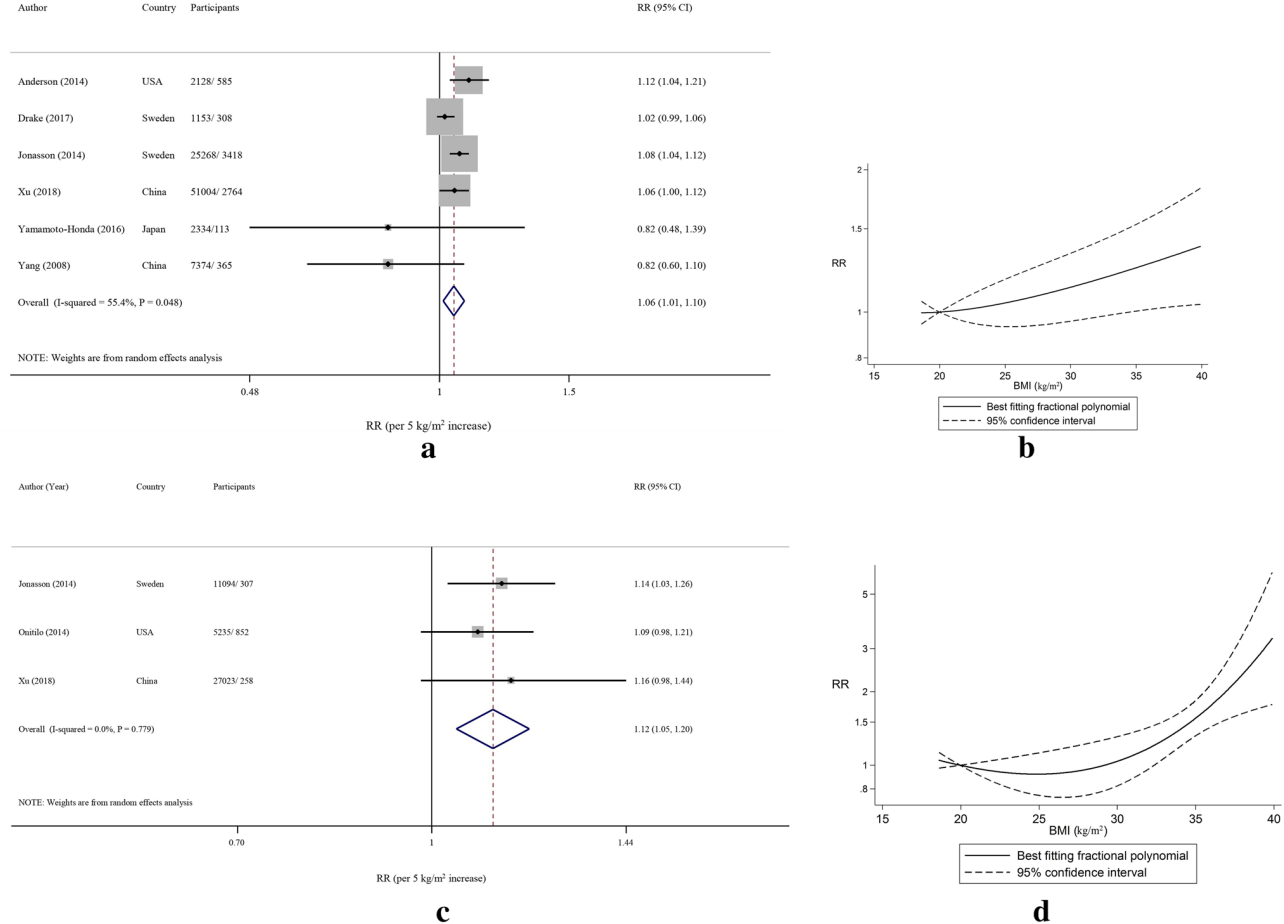
(**A**) Risk of total cancer associated with each 5-unit increase in body mass index in patients with T2D. The study-specific relative risk and 95% CI are represented by the black square and horizontal line, respectively; the area of the black square is proportional to the specific-study weight to the overall meta-analysis. The center of the open diamond presents the pooled RR and its width represents the pooled 95% CI. Weights are from the random-effects analysis. (**B**) Dose–response association of body mass index and total cancer risk in patients with T2D (*P*_non-linearity_ = 0.99). (**C**) Risk of breast cancer associated with each 5-unit increase in body mass index in patients with T2D. The study-specific relative risk and 95% CI are represented by the black square and horizontal line, respectively; the area of the black square is proportional to the specific-study weight to the overall meta-analysis. The center of the open diamond presents the pooled RR and its width represents the pooled 95% CI. Weights are from the random-effects analysis. (**D**) Dose–response association of body mass index and breast cancer risk in patients with T2D (*P*_non-linearity_ = 0.004).

Four prospective and two retrospective cohort studies^29,30,32–35^ with 89,261 participants and 7553 cases were included in the analysis of the association between BMI and the risk total cancer in patients with T2D. The analysis indicated that each 5-unit increase in BMI (kg/m^2^) was associated with a 6% higher risk of total cancer incidence (RR: 1.06, 95% CI 1.01, 1.10), with high heterogeneity, *I*^2^ = 55.4%, *P*_heterogeneity_ < 0.001 (Fig. 3A). In stratified analyses, the association was positive in most subgroups although not always statistically significant, however, there was no evidence of between subgroup heterogeneity (Table 1). The analysis of cohort studies suggested a positive monotonic association between BMI with total cancer development (*P*_non-linearity_ = 0.99) (Fig. 3B). The non-linear RRs and corresponding 95% CIs for the association between BMI and total cancer are provided in Supplementary Table S4. A significant increasing trend persisted when each study was sequentially removed from the meta-analysis (RR range: 1.04–1.08) (Supplementary Figure S1).

**Table 1:** Meta-analysis of body mass index and risk of total cancer in patients with T2D (all analyses were conducted using random-effects models).

Subgroup	Num. of studies	Meta-analysis	Heterogeneity			
		RR (95% CI)	Q statistic	I^2^ (%)	P heterogeneity^a^	P between^b^
Overall	6	1.06 (1.01, 1.10)	11.20	55.4	0.048	
**Study design**						0.89
Prospective	4	1.06 (1.00, 1.12)	10.30	70.9	0.02	
Retrospective	2	1.06 (1.00, 1.12)	0.89	0	0.35	
**Study duration**						0.15
Short duration (< 10 year)	4	1.06 (1.01, 1.11)	4.27	29.8	0.23	
Long duration (≥ 10 year)	2	1.06 (0.97, 1.16)	4.87	79.5	0.03	
**Study location**						0.25
USA	1	1.12 (1.04, 1.21)	0	–	–	
Europe	2	1.05 (0.99, 1.11)	4.9	79.8	0.03	
Asia	3	0.97 (0.80, 1.17)	3.49	42.7	0.17	
**Adjustment for confounders**						
**Age**						–
No	0	–	–	–	–	
Yes	6	1.06 (1.01, 1.10)	11.20	55.4	0.048	
**Smoking**						0.93
No	2	0.97 (0.77, 1.23)	2.66	62.5	0.10	
Yes	4	1.06 (1.00, 1.12)	8.53	64.8	0.04	
**Alcohol**						0.13
No	3	1.06 (1.01, 1.12)	3.31	39.5	0.19	
Yes	3	1.05 (0.97, 1.15)	5.61	64.4	0.06	
**Physical activity**						0.15
No	4	1.06 (1.01, 1.11)	4.27	29.8	0.23	
Yes	2	1.06 (0.97, 1.16)	4.87	79.5	0.03	

*RR* relative risk, *CI* confidence interval.

^a^p-value for heterogeneity within groups.

^b^p-value for heterogeneity between group.

### Pancreatic cancer

Two prospective cohort studies^38,40^ with 611,083 participants and 666 cases were included in the analysis of the association between BMI and the risk pancreatic cancer in patients with T2D. There was no association between a 5-unit increase in BMI (kg/m^2^) and the risk pancreatic cancer (RR: 0.97, 95% CI 0.84, 1.11) with high heterogenity, *I*^2^ = 71%, *P*_heterogeneity_ = 0.06 (Supplementary Figure S2).

### Colorectal cancer

Two prospective cohort studies^32,41^ with 2,616,417 participants and 24,827 cases were included in the analysis of the association between BMI and the risk colorectal cancer in patients with T2D. There was no association between a 5-unit increase in BMI (kg/m^2^) and the risk colorectal cancer (RR: 1.05, 95% CI 0.98, 1.13) with high heterogenity, *I*^2^ = 65.9%, *P*_heterogeneity_ = 0.09 (Supplementary Figure S3).

### Prostate cancer

Four prospective cohort studies^28,32,36,39^ with 62,477 participants and 3,387 cases were included in the analysis of the association between BMI and the risk of prostate cancer in patients with T2D. There was no association between a 5-unit increase in BMI (kg/m^2^) and the risk prostate cancer (RR: 1.02, 95% CI 0.92, 1.13) with high heterogenity, *I*^2^ = 64.4%, *P*_heterogeneity_ = 0.04 (Supplementary Figure S4). The null association persisted across all subgroups (Table 2).

**Table 2:** Meta-analysis of body mass index and risk of prostate cancer in patients with T2D (all analyses were conducted using random-effects models).

Subgroup	Num. of studies	Meta-analysis	Heterogeneity			
		RR (95% CI)	Q statistic	I^2^ (%)	P heterogeneity^a^	P between^b^
Overall	4	1.02 (0.92, 1.13)	8.46	64.6	0.04	
**Study design**						0.55
Prospective	4	1.02 (0.92, 1.13)	8.46	64.6	0.04	
**Study duration**						0.34
Short duration (< 10 year)	2	0.99 (0.91, 1.07)	0.36	0	0.55	
Long duration (≥ 10 year)	2	1.06 (0.86, 1.31)	7.20	86.1	0.01	
**Study location**						0.02
USA	1	0.96 (0.87, 1.06)	0	–	–	
Europe	2	0.99 (0.91, 1.07)	0.38	0	0.55	
Asia	1	1.19 (1.05, 1.34)	0	–	–	
**Confounders adjustment**						
**Age**						–
No	0	–	–	–	–	
Yes	4	1.02 (0.92, 1.13)	8.46	64.6	0.04	
**Smoking**						–
No	0	–	–	–	–	
Yes	4	1.02 (0.92, 1.13)	8.46	64.6	0.04	
**Alcohol**						0.005
No	3	0.98 (0.92, 1.04)	0.58	0	0.75	
Yes	1	1.19 (1.05, 1.34)	0	–	–	
**Physical activity**						0.007
No	3	0.98 (0.92, 1.04)	0	0	0.75	
Yes	1	1.19 (1.05, 1.34)	0	–	–	

*RR* relative risk, *CI* confidence interval.

^a^p-value for heterogeneity within groups.

^b^p-value for heterogeneity between group.

In the sensitivity analysis, sequential exclusion of each study did not materially change the summary estimate (RR range: 0.98–1.05) (Supplementary Figure S5).

### Breast cancer

Two prospective and one retrospective cohort study^29,32,37^ with 43,352 participants and 839 cases were included in the analysis of the association between BMI and the risk breast cancer in patients with T2D. The analysis indicated that each 5-unit increase in BMI (kg/m^2^) was associated with an 12% increased risk of breast cancer incidence (RR: 1.12, 95% CI 1.05, 1.20), with no evidence of heterogeneity, *I*^2^ = 0%, *P*_heterogeneity_ = 0.78 (Fig. 3C). The analysis suggested a positive association between BMI with breast cancer development with steeper increases in risk from a BMI around 35 (*P*_non-linearity_ = 0.004) (Fig. 3D). The RRs (95% CIs) from the non-linear dose–response analysis of BMI and breast cancer are provided in Supplementary Table S4.

### Ancilary analysis

In order to assess the potential impacts of confounding bias, the linear dose–response analyis was conducted based on crude/unadjusted risk estimates (Supplementary Figure S6).

## Discussion

In this dose–response meta-analysis of 13 cohort studies on BMI and cancer risk in patients with T2D there was a significant 6% increase in total cancer risk and a significant 12% increase in breast cancer risk per 5 units increase in BMI. We also found a potential nonlinear association between BMI and breast cancer risk with steeper increases in risk from a BMI around 35, while the association with total cancer appeared to be linear. These findings are similar to those of previous meta-analyses that reported a higher risk of total cancer^26^ and breast cancer with increasing BMI^42^. However, previous meta-analyses have focused on the risk of cancer in general population and this is to our knowledge the first study to evaluate the linear and potential non-linear relationship between BMI and site-specific cancer risk in patients with T2D.

Obesity is a well-known risk factor for both diabetes and different types of cancers (17, 54). Findings from epidemiological studies also suggest that patients with T2D have increased risk of developing several cancers including colorectal (15), pancreatic (55), liver (56), gallbladder (57), breast (14), and endometrial (58) cancer. Metabolic, hormonal and physiological imbalances associated with diabetes and obesity could explain the positive association between higher BMI and cancer risk in patients with T2D. Adiposity is accompanied by insulin resistance, which is implicated in the development of cancer and tumor cells proliferation (20). Obesity-induced oxidative stress and low-grade systemic inflammation may also contribute to the increased risk of cancer in patients with T2D and excess weight (59). In addition, obesity-induced hypoxia and migrating adipose stromal cells are the emerging mechanisms linking obesity to carcinogenesis in patients with T2D (60). Moreover, estrogens and estradiol which are mainly produced by fat tissue, can activate cell proliferation and increase DNA damage in the breast and endometrial cells; resulting in tumor progression and cancer development (61).

Some limitations may have affected the results of the current analysis. As meta-analyses of observational studies are prone to the biases that are inherent in the included studies, it is possible that confounding or reverse causation could have impacted the results, and heterogeneity between studies was also observed in several analyses. Obesity and diabetes is usually associated with other unhealthy behaviors such as sedentary lifestyle, alcohol intake, smoking, dietary habits etc., which are also related to cancer incidence. Although we cannot completely rule out the possibility that confounding could have affected the results, the associations were similar in most subgroup analyses stratified by the most important confounders (age, smoking, alcohol, and physical activity) and there was no heterogeneity between these subgroups, however, there were few studies that adjusted for dietary factors or took into account use of antidiabetic medications or diabetes duration. One exception was the positive association between BMI and total cancer, which was restricted to studies with adjustment for smoking, and not observed among studies without adjustment for smoking (although the test for heterogeneity between subgroups was not significant). Smoking is a strong risk factor for a large range of cancers (62–65) and lack of adjustment or stratification for smoking could potentially confound the association with cancer toward the null as smokers tend to have a lower BMI than non-smokers^43^. It is possible that use of certain anti-diabetic medications could have attenuated the association between BMI and some cancers in the current analysis as some studies have suggested metformin could reduce risk of some cancers^44^.

We also conducted subgroup analyses stratified by study duration, study design, geographic location, but found little indication of differences across these subgroups. Reverse causation could have affected the results because several cancers are accompanied by weight loss, however, no studies excluded early follow-up to take this into account. However, since the results were similar when studies were stratified by duration of follow-up it is less likely that this may have had a substantial impact on the results as reverse causation would have affected studies with short follow-up to a larger degree than studies with long follow-up.

For pancreatic cancer our null finding was inconsistent with a previous meta-analysis which found a positive association between higher BMI and increased risk of this cancer in general population studies^45^. The association between BMI and colorectal cancer risk was also not significant in the current analysis, however, this was based on only two studies and the the summary estimate is overlapping with the summary estimate from a meta-analysis of studies in the general population (1.05, 0.98–1.13 vs. 1.06, 1.03–1.07)^46^, and suggest further studies are needed in patients with T2D. Our null finding for BMI and overall prostate cancer risk is consistent with the results from the WCRF report, however, we were not able to look at fatal and advanced cancers, for which there is evidence of increased risk^8^. There was a limited number of studies across specific cancers and further studies are therefore needed across most cancer sites.

Although, indices of central obesity and body fat distribution may be more appropriate predictors for cancer risk than BMI^29,31,32,35,38^, we were unable to examine the association of central fatness with risk of cancer in diabetes. Another point also to be noted is that there are gender differences in the incidence of some cancers^47,48^, and we had limited possibility to conduct gender-specific analyses because most studies did not report stratified analyses. Although menopausal status is an effect modified of the association between BMI and breast cancer risk, only one study reported results stratified by menopausal status. Finally, because almost all studies included in the present study only performed a single baseline BMI assessment, we were not able to take into account potential changes in BMI that may have occurred after baseline.

The present meta-analysis has several strengths. We included only prospective and retrospective cohort studies in the current meta-analysis and this reduced the possibility that recall bias and selection bias, which to a larger degree can affect case–control studies, could explain our findings. By combining data from several studies we had better statistical power than any of the individual studies to detect an association, but there may still have been insufficient statistical power for several of the cancers investigated because of the low number of studies. We investigated the associations for total and site-specific cancers to provide a comprehensive overview of the available data. Lastly, a relatively comprehensive search strategy was developed to identify all the relevant published literature. We also conducted dose–response analyses to clarify the shape of the associations in the analyses of total cancer and breast cancer.

## Conclusions

In conclusion, this dose–response meta-analysis of cohort studies found a significant positive association between higher BMI and increased risk of total cancer and breast cancer in patients with T2D. Although, these results are limited by the low number of studies published to date, they are largely consistent with results from studies in the general population. Given the increasing global prevalence of diabetes, further high-quality prospective cohort studies are needed to evaluate the association between BMI across a larger number of cancers in patients with T2D to obtain a more complete picture of these associations.

## Methods

The Preferred Reporting Items for Systematic reviews and Meta-Analyses (PRISMA) statement^49^ and Meta-analyses Of Observational Studies in Epidemiology (MOOSE) guideline^50^ were followed as guidance for reporting this meta-analysis (Supplementary Table S5). The study protocol was registered in the international prospective register of systematic reviews database (http://www.crd.york.ac.uk/PROSPERO, registration No: CRD 42019132981).

### Data sources and searches

We searched PubMed, Scopus, and Medline databases to September 08, 2020 without any language restrictions for cohort studies in adult humans on the relationship between BMI and incidence of overall and site-specific cancers. The search query was as follows: (obesity OR body mass index) AND (cancer OR neoplasm OR carcinoma) AND (cohort OR follow-up OR prospective) AND (diabetes). The detailed search strategy is provided in Supplementary Table S6. The bibliographies of relevant articles were also searched for potential further publications.

### Dealing with missing data

We contacted the authors of the Malmo Diet and Cancer Study (MDCS)^34^ and Iowa Women's Health Study (IWH)^33^ to obtain the risk estimates of cancer in patients with T2D and data about category-specific numbers of participants/cases across categories of BMI.

### Study selection

The following inclusion criteria were used to find the potential eligible articles: the studies had to be (1) based on prospective/retrospective cohort, or nested case–control study design, (2) conducted in adults with existing type 2 diabetes, (3) use self-reported or measured BMI as exposure and incidence of overall or site-specific cancers as the outcome, and (4) reporting adjusted risk estimates (odds ratios, risk ratios, or hazard ratios) with their 95% confidence intervals (CIs) and the numbers of cases and person-years or participants/non-cases across three or more quantitative categories of BMI, or reporting sufficient data to calculate these values. Studies that presented results on a continuous scale per standard deviation (SD) or unit increment in BMI were also included. Studies were excluded if they had retrospective case–control or cross-sectional design. In case of studies that did not provide sufficient information to calculate effect sizes, corresponding authors were contacted, and of those, two studies provided additional data from their studies^33,34^. Two authors (SS and Sh A) independently screened the title and abstract of all articles retrieved. Any disagreements were resolved by consensus with the third author (AJ). The Cohen’s κ coefficient was calculated to indicate the interrater agreement at the abstract and full-text screening stages.

### Data extraction and quality assessment

One author (SS), by using a pre-defined data-collection form, extracted the following data from included studied, which was double checked by another author (AJ): the first author’s name, publication year, study location, study design (prospective/ retrospective cohort), follow-up duration, gender and age of participants, number of participants and cases, the criteria used for type 2 diabetes diagnosis, type of cancer (overall or specific site), the method of exposure assessment (self-reported or measured), corresponding risk estimates with 95% CIs in fully adjusted models, and the confounding variables that were adjusted for. The crude crude/unadjusted risk estimates were calculated for each cancer in a sensitivity analysis to assess the potential impacts of confounding bias. The study quality was evaluated with the use of a 9-star Newcastle– Ottawa Scale^51^. This scale is based on the following items: representativeness of the study populations, exposure assessment, adjustment for potential confounding factors, assessment of outcome, and length and adequacy of follow-up.

### Data synthesis and analysis

Estimates of the relative risk (RR) and its 95% CIs such as hazard ratios and odds ratios were considered as equal to RRs given the rarity of cancer cases in patients with T2D. Summary RRs and 95% CIs of total and site-specific cancer were estimated for each 5-unit increase in BMI with the use of a random-effects model^52^. The method proposed by Greenland and Longnecker^53^ was used for the linear dose–response analysis. The distribution of cases and participants/non-cases or person-years and adjusted risk estimates with their 95% CI for different categories of BMI were requisite input for using this method. The median point of BMI in each category was used when it was provided in the articles. For studies that reported the BMI categories in ranges we estimated the upper and lower cut-off value for open-ended categories (the first and the last categories) by using the width of the adjacent category. For studies in which the risk estimates were reported for a 1-SD or a 3- or 4-unit increment in BMI, the reported risk estimates were recalculated for a 5-unit increment in BMI. For those studies in which the lowest BMI category was not the reference category, the effect sizes were recalculated assuming the lowest category as reference, by the method described by Hamling^54^. If the study reported risk estimates in different age or gender subgroups, these risk estimates were pooled by a fixed-effects model to generate an overall estimate before inclusion in the main analysis. The Cochrane’s Q test and *I*^2^ statistics were used to evaluate the heterogeneity among the included studies. The following cut-off points were concidered to interperet the *I*^2^ statistic: < 25% (low heterogeneity), 25–50% (moderate heterogeneity), > 50–75% (high heterogeneity) and > 75% (severe heterogeneity)^55^. Subgroup analyses and meta-regression were performed by the study design (prospective vs/retrospective cohort), study location (USA, Europe, and Asia), study duration (cut-off point: 10 years), and confounders adjusted (age, smoking, alcohol, physical activity) to detect the potential sources of heterogeneity. When there was at least four studies included in the analysis we also conducted sensitivity analyses excluding one study at a time to examine the impact of each study on the summary estimate. Publication bias was not evaluated owing to the small number of studies (less than 10 studies)^56^. Nonlinear analyses were conducted using fractional polynomial models and we determined the best fitting second order polynomial regression model, defined as the one with the lowest deviance^57^. A likelihood ratio test was used to assess the difference between the nonlinear and linear models to test for nonlinearity^57^. All statistical analyses were conducted using STATA software, version 13.0 (Stata Corp, College Station, Texas, USA).

## Supplementary Information

Supplementary Information

## Data Availability

The datasets generated during and/or analysed during the current study are available from the corresponding author on reasonable request.
