# Supplementary material for: Body mass index and cancer risk in patients with type 2 diabetes: a dose–response meta-analysis of cohort studies
Source: Sci Rep. 2021 Jan 28;11:2479. doi: 10.1038/s41598-021-81671-0 (PMC7844243; doi:10.1038/s41598-021-81671-0)
Supplement: Supplementary file 1 — Supplementary Information [file 41598_2021_81671_MOESM1_ESM.docx]

Body Mass Index and Cancer Risk in Patients with Type 2 Diabetes: Dose–Response Meta-analysis of Cohort Studies

Sepideh Soltani, Shima Abdollahi, Dagfinn Aune, Ahmad Jayedi

**Legends to supplementary Tables**

**Supplementary Table S1.** Excluded studies with reasons.

**Supplementary Table S2.** Characteristics of cohort studies that investigated the association between body mass index and total and site-specifc cancer in patients with type 2 diabets.

**Supplementary Table S3.** Study quality and risk of bias assessment using New Castle-Ottawa Scale (NOS) tool.

**Supplementary Table S4.** Non-linear relative risks and theirs 95% CIs for the association between BMI and total and breast cancer

**Supplementary Table S5.** MOOSE statement checklist

**Supplementary Table S6.** Literature search strategy.

| **Supplementary Table S1.** Excluded studies with reasons. |
| --- |
| **BMI in non diabetic population was considered as reference categories (n=4)**   1. Bae, W. J. *et al.* Influence of diabetes on the risk of urothelial cancer according to body mass index: a 10-year nationwide population-based observational study. *Journal of Cancer* **9**, 488-493, doi:10.7150/jca.22107 (2018). 2. Lai, G., Park, Y., Hartge, P., Hollenbeck, A. & Freedman, N. The Association Between Self-Reported Diabetes and Cancer Incidence in the NIH-AARP Diet and Health Study. *J Clin Endocrinol Metab* **98**, E497-E502, doi:10.1210/jc.2012-3335 (2013). 3. Luo, J. *et al.* Body mass index, physical activity and the risk of pancreatic cancer in relation to smoking status and history of diabetes: A large-scale population-based cohort study in Japan - The JPHC study. *Cancer Causes and Control* **18**, 603-612, doi:10.1007/s10552-007-9002-z (2007). 4. Seow, A., Yuan, J. M., Koh, W. P., Lee, H. P. & Yu, M. C. Diabetes mellitus and risk of colorectal cancer in the Singapore Chinese Health Study. *J Natl Cancer Inst* **98**, 135-138, doi:10.1093/jnci/djj015 (2006). |
| **Not relevant exposure (n=15)**  1 Feng, X. *et al.* The association between fasting blood glucose and the risk of primary liver cancer in Chinese males: a population-based prospective study. *British journal of cancer* **117**, 1405-1411, doi:10.1038/bjc.2017.296 (2017).  2 Joh, H. K., Willett, W. C. & Cho, E. Type 2 diabetes and the risk of renal cell cancer in women. *Diabetes care* **34**, 1552-1556, doi:10.2337/dc11-0132 (2011).  3 Kasper, J. S., Liu, Y. & Giovannucci, E. Diabetes mellitus and risk of prostate cancer in the health professionals follow-up study. *International journal of cancer* **124**, 1398-1403, doi:10.1002/ijc.24044 (2009).  4 Kitahara, C. M. *et al.* Physical activity, diabetes, and thyroid cancer risk: a pooled analysis of five prospective studies. *Cancer causes & control : CCC* **23**, 463-471, doi:10.1007/s10552-012-9896-y (2012).  5 La Vecchia, C., Negri, E., Decarli, A. & Franceschi, S. Diabetes mellitus and colorectal cancer risk. *Cancer epidemiology, biomarkers & prevention : a publication of the American Association for Cancer Research, cosponsored by the American Society of Preventive Oncology* **6**, 1007-1010 (1997).  6 Le Guillou, A. *et al.* [Is cancer incidence different between type 2 diabetes patients compared to non-diabetics in hemodialysis? A study from the REIN registry]. *Nephrologie & therapeutique* **14**, 142-147, doi:10.1016/j.nephro.2017.02.018 (2018).  7 Leitzmann, M. F. *et al.* Diabetes mellitus and prostate cancer risk in the Prostate, Lung, Colorectal, and Ovarian Cancer Screening Trial. *Cancer causes & control : CCC* **19**, 1267-1276, doi:10.1007/s10552-008-9198-6 (2008).  8 Michels, K. B. *et al.* Type 2 diabetes and subsequent incidence of breast cancer in the Nurses' Health Study. *Diabetes care* **26**, 1752-1758 (2003).  9 Minicozzi, P. *et al.* High fasting blood glucose and obesity significantly and independently increase risk of breast cancer death in hormone receptor-positive disease. *European journal of cancer (Oxford, England : 1990)* **49**, 3881-3888, doi:10.1016/j.ejca.2013.08.004 (2013).  10 Moreira, D. M. *et al.* The association of diabetes mellitus and high-grade prostate cancer in a multiethnic biopsy series. *Cancer causes & control : CCC* **22**, 977-983, doi:10.1007/s10552-011-9770-3 (2011).  11 Palmer, J. R., Castro-Webb, N., Bertrand, K., Bethea, T. N. & Denis, G. V. Type II Diabetes and Incidence of Estrogen Receptor Negative Breast Cancer in African American Women. *Cancer research* **77**, 6462-6469, doi:10.1158/0008-5472.can-17-1903 (2017).  12 Pang, Y. *et al.* Central adiposity in relation to risk of liver cancer in Chinese adults: A prospective study of 0.5 million people. *International journal of cancer*, doi:10.1002/ijc.32148 (2019).  13 Prizment, A. E., Anderson, K. E., Yuan, J. M. & Folsom, A. R. Diabetes and risk of bladder cancer among postmenopausal women in the Iowa Women's Health Study. *Cancer causes & control : CCC* **24**, 603-608, doi:10.1007/s10552-012-0143-3 (2013).  14 Simon, T. G. *et al.* Diabetes, metabolic comorbidities, and risk of hepatocellular carcinoma: Results from two prospective cohort studies. *Hepatology (Baltimore, Md.)* **67**, 1797-1806, doi:10.1002/hep.29660 (2018).  15 Vecchia, C. L. A., Negri, E., Decarli, A. & Franceschi, S. Diabetes mellitus and the risk of primary liver cancer. *International journal of cancer* **73**, 204-207, doi:10.1002/(SICI)1097-0215(19971009)73:2<204::AID-IJC7>3.0.CO;2-# (1997). |
| **Assessed cancer recurrence (n=2)**  1 Mu, L. *et al.* Type 2 diabetes, insulin treatment and prognosis of breast cancer. *Diabetes/metabolism research and reviews* **33**, doi:10.1002/dmrr.2823 (2017).  2 Onitilo, A. A. *et al.* Increased risk of colon cancer in men in the pre-diabetes phase. *PloS one* **8**, e70426, doi:10.1371/journal.pone.0070426 (2013). |
| **Not relevant outcome (n=3)**  1 Budzynska, K. *et al.* Diabetes mellitus and hyperglycemia control on the risk of colorectal adenomatous polyps: a retrospective cohort study. *BMC family practice* **19**, 145, doi:10.1186/s12875-018-0835-1 (2018).  2 Suh, S. *et al.* Korean type 2 diabetes patients have multiple adenomatous polyps compared to non-diabetic controls. *Journal of Korean medical science* **26**, 1196-1200, doi:10.3346/jkms.2011.26.9.1196 (2011).  3 Cho, Y. H. *et al.* Does metformin affect the incidence of colonic polyps and adenomas in patients with type 2 diabetes mellitus? *Intestinal research* **12**, 139-145, doi:10.5217/ir.2014.12.2.139 (2014). |
| **Case-control study (n=2)**  1 Dabrowski, M., Szymanska-Garbacz, E., Miszczyszyn, Z., Derezinski, T. & Czupryniak, L. Risk factors for cancer development in type 2 diabetes: A retrospective case-control study. *BMC cancer* **16**, 785, doi:10.1186/s12885-016-2836-6 (2016).  2 Shoff, S. M. & Newcomb, P. A. Diabetes, body size, and risk of endometrial cancer. *American journal of epidemiology* **148**, 234-240 (1998). |
| **Conducted in non-diabetic patients (n=2)**  1 Darbinian, J. A. *et al.* Glycemic status and risk of prostate cancer. *Cancer Epidemiology Biomarkers and Prevention* **17**, 628-635, doi:10.1158/1055-9965.EPI-07-2610 (2008).  2 Hu, F. *et al.* Prospective Study of Adult Onset Diabetes Mellitus (Type 2) and Risk of Colorectal Cancer in Women. *J Natl Cancer Inst* **91**, 542-547 (1999). |
| **Without relevant data (n=3)**  1 Lukasiewicz, D., Chodorowska, M. & Jakubowska, I. [Obesity as a factor in the development of cancer in type 2 diabetes]. *Polski merkuriusz lekarski : organ Polskiego Towarzystwa Lekarskiego* **38**, 135-139 (2015).  2 Schrijnders, D. *et al.* Sex differences in obesity related cancer incidence in relation to type 2 diabetes diagnosis (ZODIAC-49). *PloS one* **13**, e0190870, doi:10.1371/journal.pone.0190870 (2018).  3 Kong, A. P. *et al.* Severe hypoglycemia identifies vulnerable patients with type 2 diabetes at risk for premature death and all-site cancer: the Hong Kong diabetes registry. *Diabetes care* **37**, 1024-1031, doi:10.2337/dc13-2507 (2014). |
| **Two categories of exposure (n=10)**  1 Brodovicz, K. *et al.* Impact of diabetes duration and chronic pancreatitis on the association between type 2 diabetes and pancreatic cancer risk. *Diabetes Obes Metab* **14**, 1123-1128, doi:10.1111/j.1463-1326.2012.01667.x (2012).  2 Cleveland, R. J. *et al.* The association of diabetes with breast cancer incidence and mortality in the Long Island Breast Cancer Study Project. *Cancer causes & control : CCC* **23**, 1193-1203, doi:10.1007/s10552-012-9989-7 (2012).  3 Er, K.-C. *et al.* Effect of glycemic control on the risk of pancreatic cancer: A nationwide cohort study. *Medicine (Baltimore)* **95**, e3921, doi:10.1097/MD.0000000000003921 (2016).  4 Friberg, E., Mantzoros, C. S. & Wolk, A. Diabetes and risk of endometrial cancer: a population-based prospective cohort study. *Cancer epidemiology, biomarkers & prevention : a publication of the American Association for Cancer Research, cosponsored by the American Society of Preventive Oncology* **16**, 276-280, doi:10.1158/1055-9965.epi-06-0751 (2007).  5 Hense, H. W., Kajüter, H., Wellmann, J. & Batzler, W. U. Cancer incidence in type 2 diabetes patients - First results from a feasibility study of the D2C cohort. *Diabetology and Metabolic Syndrome* **3**, doi:10.1186/1758-5996-3-15 (2011).  6 Luo, J. *et al.* Association between diabetes, diabetes treatment and risk of developing endometrial cancer. *British journal of cancer* **111**, 1432-1439, doi:10.1038/bjc.2014.407 (2014).  7 Maskarinec, G. *et al.* Type II Diabetes, Obesity, and Breast Cancer Risk: The Multiethnic Cohort. *Cancer epidemiology, biomarkers & prevention : a publication of the American Association for Cancer Research, cosponsored by the American Society of Preventive Oncology* **26**, 854-861, doi:10.1158/1055-9965.epi-16-0789 (2017).  8 Moe, B. & Nilsen, T. I. Cancer risk in people with diabetes: Does physical activity and adiposity modify the association? Prospective data from the HUNT Study, Norway. *Journal of diabetes and its complications* **29**, 176-179, doi:10.1016/j.jdiacomp.2014.12.001 (2015).  9 Munigala, S., Singh, A., Gelrud, A. & Agarwal, B. Predictors for Pancreatic Cancer Diagnosis Following New-Onset Diabetes Mellitus. *Clinical and translational gastroenterology* **6**, e118, doi:10.1038/ctg.2015.44 (2015).  10 Samanic, C. *et al.* Obesity and cancer risk among white and black United States veterans. *Cancer causes & control : CCC* **15**, 35-43, doi:10.1023/B:CACO.0000016573.79453.ba (2004). |
| **Unadjusted hazard ratios (n=2)**  1 Bronsveld, H. K. *et al.* Trends in breast cancer incidence among women with type-2 diabetes in British general practice. *Primary care diabetes* **11**, 373-382, doi:10.1016/j.pcd.2017.02.001 (2017).  2 Duan, D. *et al.* Does body mass index and adult height influence cancer incidence among Chinese living with incident type 2 diabetes? *Cancer epidemiology* **53**, 187-194, doi:10.1016/j.canep.2018.02.006 (2018). |

| **Supplementary Table S2.** Characteristics of cohort studies that investigated the association between body mass index and total and specific-site cancer in patients with type 2 diabetes and were eligible for inclusion in the meta-analysis. | | | | | | | |
| --- | --- | --- | --- | --- | --- | --- | --- |
| Author, Year (ref) | Study design / Country (Follow-up duration) years | Participants: number of cases | Sex (Age) | Exposure / outcome assessment | BMI cut-off values or midpoints | RR (95% CI) | Covariates |
| **Total cancer** |  |  |  |  |  |  |  |
| Anderson, 2001 ^33^ | Prospective / USA (28) | 2128: 585 | Female (55-69) | Measured/ National Cancer Institute’s Surveillance | 20.91  23.98  26.33  29.97  36.18 | 1.00  0.94 (0.63-1.41)  1.46 (1.04-2.05)  1.27 (0.93-1.75)  1.50 (1.11-2.01) | Age, smoking status, alcohol consumption, physical activity level. |
| Drake, 2017 ^34^ | Prospective/ Sweden (17) | 1153: 308 | Both (44-73) | Measured/ Swedish Cause of Death registry | <25  25- 29.9  >30 | 1.00  0.82 (0.61, 1.12)  1.09 (0.78, 1.51) | Age, height, smoking status, physical activity level, alcohol consumption, educational level, past food habit change, hypertension, lipid-lowering drugs, and family history of cancer |
| Jonasson, 2014 ^32^ | Prospective/ Sweden (8.6) | 25,268: 3418 | Both (30- 90) | Measured/ Cancer register | 18.5-24.9  25-29.9  >30 | 1.00  1.13 (1.03, 1.23)  1.22 (1.11, 1.34) | Age, HbA1c, smoking, diabetes duration, diabetes medication |
| Xu, 2018 ^29^ | Retrospective / China (4.28) | 51,004: 2764 | Both (61.3) | Self-reported/ Shanghai Cancer Registry | <18.5  18.5-24.9  25-29.9  >30 | 1.00  0.90 (0.72, 1.13)  0.94 (0.74, 1.20)  1.12 (0.82, 1.53) | Age at diagnosis of diabetes, comorbidity of hypertension , and family history of diabetes |
| Yamamoto-Honda, 2016^30^ | Retrospective / Japan (5.1) | 2334: 113 | Both (>18) | Measured/ Cancer Registry | <22  22-25  >25 | 1.00  0.98 (0.63, 1.54)  0.75 (0.39, 1.45) | Age, smoking and frequency of alcohol consumption |
| Yang, 2008^35^ | Prospective / China (5.45) | 7374: 365 | Both (57) | Measured/ National registry | <24  24-27.5  >27 | 1.00  0.69 (0.53, 0.92)  1.01 (0.64, 1.56) | Age, sex, high-density lipoprotein cholesterol, total cholesterol, triglyceride, white blood cell count and smoking status, duration of diabetes, glycated hemoglobin, spot urine albumin: creatinine ratio, glomerular filtration rate, systolic/diastolic blood pressure, peripheral arterial disease, sensory neuropathy, retinopathy and baseline drug use (lipid-lowering drugs, ACE inhibitors or angiotensin II receptor blockers and insulin) |
| **Pancreatic cancer** |  |  |  |  |  |  |  |
| Boursi, 2017 ^36^ | Retrospective/ UK (3) | 109,385: 390 | Both (>35) | Measured/ Health Improvement Network database | Per 5 unit | 0.90 (0.81, 1.00) | Age, change in BMI per year, smoking, antidiabetic medications and PPIs, HbA1C, hemoglobin, total cholesterol, creatinine and alkaline phosphatase. |
| Stolzenberg-Solomon, 2013^37^ | Prospective / USA (10.5) | 501,702: 2,122 | Both (50-71) | Self-reported/ Cancer registry | Per 5 unit | 1.04 (0.93, 1.16) | Age, smoking, energy intake, energy-adjusted total fat, and sex |
| Colorectal cancer |  |  |  |  |  |  |  |
| Jonasson, 2014 ^32^ | Prospective/ Sweden (8.6) | 25,268: 591 | Both (30- 90) | Measured/ Cancer register | 18.5-24.9  25-29.9  >30 | 1.00  1.35 (1.08, 1.69)  1.52 (1.20, 1.93) | Age, HbA1c, smoking, diabetes duration, diabetes medication |
| Lee, 2019 ^41^ | Prospective/ Korea (5.4) | 2,591,149: 24,236 | Both (>20) | Self-reported/ Cancer register | <18.5  18.5- 23  23- 25  25-30  >30 | 1.00  1.06 (0.94, 1.21)  1.05 (0.93, 1.19)  1.10 (0.97, 1.24)  1.17 (1.03, 1.34) | Age, sex, BMI, smoking, alcohol consumption, exercise, income, hypertension, dyslipidemia, history of stroke and cardiovascular events, nephropathy, retinopathy, T2DM medications, and T2DM disease duration |
| **Prostate cancer** |  |  |  |  |  |  |  |
| Choi, 2016 ^39^ | Prospective / Korea (10) | 16,679: 1171 | Male (40-64) | Measured/ National Health Insurance System | <18.5  18.5- 22.9  23- 24.9  >25 | 1.00  1.89 (1.06, 3.36)  2.02 (1.13, 3.58)  2.18 (1.23, 3.85) | Age, hypertension, dyslipidemia, smoking status, alcohol consumption, and exercise |
| Hendriks, 2018 ^40^ | Prospective / Netherlands (3.1) | 25,811: 170 | Male (64) | Measured/ Netherlands Cancer registry | 18.5- 25  25- 30  30- 35 | 1.00  1.07 (0.69, 1.66)  1.15 (0.71, 1.86) | Age, diabetes duration, HbA1c, serum creatinine, smoking status and the use of metformin, sulfonylurea derivatives and/or insulin. |
| Jonasson, 2014 ^32^ | Prospective/ Sweden (8.6) | 25,268: 736 | Both (30- 90) | Measured/ Cancer register | 18.5-24.9  25-29.9  >30 | 1.00  1.13 (0.94, 1.36)  1.01 (0.81, 1.25) | Age, HbA1c, smoking, diabetes duration, diabetes medication |
| Onitilo, 2013 ^28^ | Prospective / USA (15) | 5813: 1310 | Male (62.9) | Measured/ Cancer registry | <25  25- 29.9  >30 | 1.00  0.85 (0.71, 1.02)  0.93 (0.76, 1.13) | Age, birth year, smoking, diabetes diagnosis period, visit frequency before and after diabetes diagnosis, insurance status, renal disease, cardiovascular disease, coronary heart disease. |
| **Breast cancer** |  |  |  |  |  |  |  |
| Jonasson, 2014 ^3^ | Prospective/ Sweden (8.6) | 25,268 : 307 | Both (30- 90) | Measured/ Cancer register | 18.5-24.9  25-29.9  >30 | 1.00  0.95 (0.69, 1.29)  1.30 (0.97, 1.75) | Age, HbA1c, smoking, diabetes duration, diabetes medication |
| Onitilo, 2014 ^38^ | Prospective/ USA (15) | 5235: 852 | Female (>30) | Measured/ Cancer registry | <25  25- 29.9  >30 | 1.00  0.94 (0.77, 1.14)  1.19 (0.96, 1.46) | Age, birth year, smoking, diabetes diagnosis period, visit frequency before and after diabetes diagnosis, insurance status, renal disease, cardiovascular disease, coronary heart disease. |
| Xu, 2018^29^ | Retrospective / China (4.28) | 51,004: 258 | Both (61.3) | Self-reported/ Shanghai Cancer Registry | <18.5  18.5-24.9  25-29.9  >30 | 1.00  0.94 (0.38, 2.32)  1.07 (0.42, 2.72)  1.56 (0.57, 4.26) | Age at diagnosis of diabetes, comorbidity of hypertension , and family history of diabetes |

**Supplementary Table S3.** Study quality and risk of bias assessment using New Castle-Ottawa Scale (NOS) tool

| Author, Year | Selection | | | | Comparability | | Outcome | | | Score | Quality |
| --- | --- | --- | --- | --- | --- | --- | --- | --- | --- | --- | --- |
|  | 1 | 2 | 3 | 4 | 1 | 2 | 1 | 2 | 3 |  |  |
| Anderson, 2001 ^33^ | * | * |  | * | * |  | * | * | * | 7 | Good |
| Boursi, 2017 ^36^ | * | * | * | * | * | * | * |  | * | 8 | Good |
| Choi, 2016 ^39^ | * | * | * | * | * | * | * | * | * | 9 | Good |
| Drake, 2017 ^34^ | * | * | * | * | * | * | * | * | * | 9 | Good |
| Hendriks, 2018 ^40^ | * | * | * | * | * |  | * |  | * | 7 | Good |
| Jonasson, 2014 ^32^ | * | * | * | * | * |  | * | * | * | 8 | Good |
| Lee, 2019 ^41^ | * | * |  | * | * | * | * |  |  | 6 | Moderate |
| Onitilo, 2014 ^28^ | * | * | * | * | * |  | * | * |  | 7 | Good |
| Onitilo, 2013 ^38^ | * | * | * | * | * |  | * | * |  | 7 | Good |
| Stolzenberg-Solomon, 2013 ^37^ | * | * |  | * | * |  | * | * |  | 6 | Moderate |
| Xu, 2018 ^29^ | * | * |  | * | * |  | * |  | * | 6 | Moderate |
| Yamamoto-Honda, 2016 ^30^ |  | * | * | * | * |  | * |  |  | 5 | Moderate |
| Yang, 2008 ^35^ | * | * | * | * | * |  | * |  |  | 6 | Moderate |

| **Supplementary Table S4.** Non-linear relative risks and theirs 95% CIs for the association between BMI and total and breast cancer | | |
| --- | --- | --- |
| BMI level | RR (95% CI) Total cancer | RR (95% CI) Breast cancer |
| 18.5 | 1.00 (0.94-1.06) | 1.05 (0.97-1.14) |
| 20.0 | 1.00 | 1.00 |
| 22.5 | 1.02 (0.95-1.09) | 0.94 (0.83-1.06) |
| 25.0 | 1.05 (0.93-1.17) | 0.92 (0.75-1.12) |
| 27.5 | 1.08 (0.94-1.25) | 0.95 (0.75-1.21) |
| 30.0 | 1.13 (0.96-1.33) | 1.04 (0.83-1.31) |
| 32.5 | 1.18 (0.98-1.42) | 1.22 (1.02-1.47) |
| 35.0 | 1.24 (1.00-1.53) | 1.56 (1.32-1.84) |
| 37.5 | 1.31 (1.02-1.67) | 2.18 (1.59-3.00) |
| 40.0 | 1.38 (1.04-1.84) | 3.39 (1.78-6.44) |
| P_nonlinearity_ | 0.99 | 0.004 |
| RR: relative risk | | |

| **Supplementary Table S5. MOOSE statement checklist** | |
| --- | --- |
| **Reporting Criteria** | **pages** |
| Reporting of Background |  |
| Problem definition | 3 |
| Hypothesis statement | 4 |
| Description of Study Outcome(s) | 12 |
| Type of exposure or intervention used | 12 |
| Type of study design used | 12 |
| Study population | 12 |
| **Reporting of Search Strategy** |  |
| Qualifications of searchers | 12 |
| Search strategy, including time period included in the synthesis and keywords | 12 |
| Effort to include all available studies, including contact with authors | 12 & 13 |
| Databases and registries searched | 11 & 12 |
| Use of hand searching (eg, reference lists of obtained articles) | 12 |
| List of citations located and those excluded, including justification | 5 & Figure 1 |
| Method for addressing articles published in languages other than English | -- |
| Method of handling abstracts and unpublished studies | -- |
| Description of any contact with authors | 13 |
| **Reporting of Methods** |  |
| Description of relevance or appropriateness of studies assembled for assessing the hypothesis to be tested | 11 & 12 |
| Rationale for the selection and coding of data | 14 |
| Documentation of how data were classified and coded | 14 |
| Assessment of confounding | 14 |
| Assessment of study quality | 13 |
| Assessment of heterogeneity | 14 |
| Description of statistical methods in sufficient detail to be replicated | 14 & 15 |
| Provision of appropriate tables and graphics | 5-7 |
| **Reporting of Results** |  |
| Table giving descriptive information for each study included | 21& 222 & Supplementary files |
| Results of sensitivity testing (eg, subgroup analysis) | 21& 222 & Supplementary files |
| **Reporting of Discussion** |  |
| Quantitative assessment of bias (eg, publication bias) | 9 |
| Assessment of quality of included studies | 9 |
| **Reporting of Conclusions** |  |
| Consideration of alternative explanations for observed results | 11 |
| Generalization of the conclusions | 11 |
| Guidelines for future research | 11 |
| Disclosure of funding source | 11 |

| **Supplementary Table S6.** Literature search strategy |
| --- |
| Medline |
| (Diabetes Mellitus/) OR (Diabetes Mellitus OR hyperglycemia OR diabet*).ti,ab |
| (*Observational OR *Follow up OR *Follow-up OR *Prospective OR *retrospective OR Nested case-control OR Relative risk OR Risk ratio OR Hazard ratio OR odds ratio OR longitudinal OR *prospectively OR *cohort OR *observations).ti,ab. OR (Cohort Studies/ OR Incidence/ OR longitudinal Studies/ OR Epidemiologic Studies/ OR *prospective studies/ OR *longitudinal studies/) |
| (obesity or obese or adiposity or body size or body mass index or BMI or body mass).ti,ab OR  (Obesity/ OR Adiposity/ OR Body Size/ OR Body Mass Index/) |
| (*cancer* or neoplasm* or carcinoma* or tumo?r* or adenocarcinoma* or oncolog* or malignan* or neoplasia).ti,ab.) OR (neoplasms/ or carcinoma/).ti,ab OR (“Non-Hodgkin lymphoma” OR “Hodgkin disease" OR lymphoma OR leukemia).ti,ab |
| Scopus |
| (TITLE-ABS (“Diabetes Mellitus” OR hyperglycemia OR diabet*) |
| (TITLE-ABS (*Observational OR “*Follow up” OR *Follow-up OR *Prospective OR *retrorospective OR “Nested case-control” OR “Relative risk” OR “Risk ratio” OR “Hazard ratio” OR “odds ratio” OR longitudinal OR *prospectively OR *cohort OR *observations) |
| (TITLE-ABS (obesity or obese or adiposity or “body size” or “body mass index” or “BMI” or “body mass”) |
| (TITLE-ABS (*cancer* or neoplasm* or carcinoma* or tumo?r* OR adenocarcinoma* OR oncolog* OR malignan* OR neoplasia OR “Non-Hodgkin lymphoma” or “Hodgkin disease" or lymphoma or leukemia) |
| Pubmed |
| *Observational[tiab] OR “*Follow up”[tiab] OR “*Follow-up” [tiab] OR *Prospective[tiab] OR *retrorospective [tiab] OR “Nested case-control” [tiab] OR “Relative risk” [tiab] OR “Risk ratio” [tiab] OR “Hazard ratio” [tiab] OR “odds ratio” [tiab] OR longitudinal[tiab] OR *prospectively[tiab] OR *cohort[tiab] OR *observations[tiab] OR *longitudinal studies [tiab] OR Incidence[tiab] OR “Cohort Studies” [mesh terms] OR Incidence [mesh terms] OR “longitudinal Studies” [mesh terms] OR “Epidemiologic Studies” [mesh terms] OR “*prospective studies” [mesh terms] |
| Diabetes Mellitus[mesh terms] OR “Diabetes Mellitus” [tiab]OR hyperglycemia [tiab]OR diabet*[tiab] |
| obesity[tiab] OR obese[tiab] OR adiposity[tiab] OR “body size”[tiab] OR “body mass index” [tiab] OR “BMI” [tiab] OR “body mass” [tiab] OR Obesity [mesh terms] OR Adiposity [mesh terms] OR Body Size [mesh terms] OR Body Mass Index [mesh terms] |
| *cancer*[tiab] OR neoplasm*[tiab] OR carcinoma*[tiab] OR tumo?r*[tiab] OR adenocarcinoma*[tiab] OR oncolog*[tiab] OR malignan*[tiab] OR neoplasia[tiab] OR neoplasms [mesh terms] OR carcinoma [mesh terms] OR “Non-Hodgkin lymphoma” [tiab] OR “Hodgkin disease"[tiab] OR lymphoma[tiab] OR leukemia[tiab] OR lymphoma [mesh terms] OR leukemia [mesh terms] |

**Legends to supplementary Figures**

**Supplementary Figure S1.** Sensitivity analysis using the fixed-effects model for risk of total cancer in patients with T2D

**Supplementary Figure S2.** Risk of pancreatic cancer associated with each 5-unit increase in body mass index in patients with T2D. The study-specific relative risk and 95 % CI are represented by the black square and horizontal line, respectively; the area of the black square is proportional to the specific-study weight to the overall meta-analysis. The center of the open diamond presents the pooled RR and its width represents the pooled 95 % CI. Weights are from the random-effects analysis

**Supplementary Figure S3**. Risk of colorectal cancer associated with each 5-unit increase in body mass index in patients with T2D. The study-specific relative risk and 95 % CI are represented by the black square and horizontal line, respectively; the area of the black square is proportional to the specific-study weight to the overall meta-analysis. The center of the open diamond presents the pooled RR and its width represents the pooled 95 % CI. Weights are from the random-effects analysis

**Supplementary Figure S4**. Risk of prostate cancer associated with each 5-unit increase in body mass index in patients with T2D. The study-specific relative risk and 95 % CI are represented by the black square and horizontal line, respectively; the area of the black square is proportional to the specific-study weight to the overall meta-analysis. The center of the open diamond presents the pooled RR and its width represents the pooled 95 % CI. Weights are from the random-effects analysis.

**Supplementary Figure S5.** Sensitivity analysis using the fixed-effects model for risk of prostate cancer in patients with T2D

**Supplementary Figure S6.** Summary crude/unadjusted risk estimates by overall and cancer sites in per 5-unit increase in body mass index in patients with T2D.


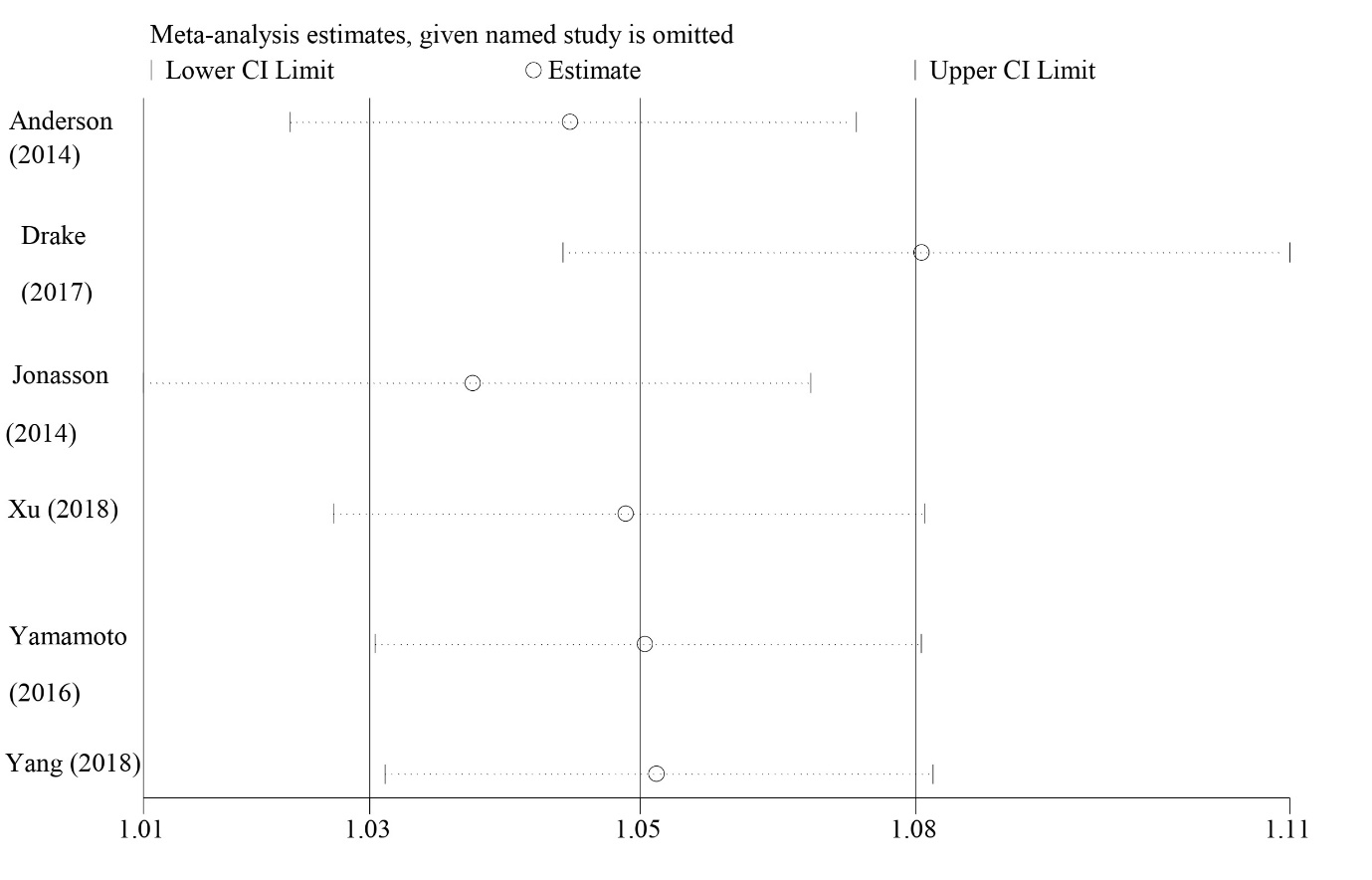


**Supplementary Figure S1.** Sensitivity analysis using the fixed-effects model for risk of total cancer in patients with T2D


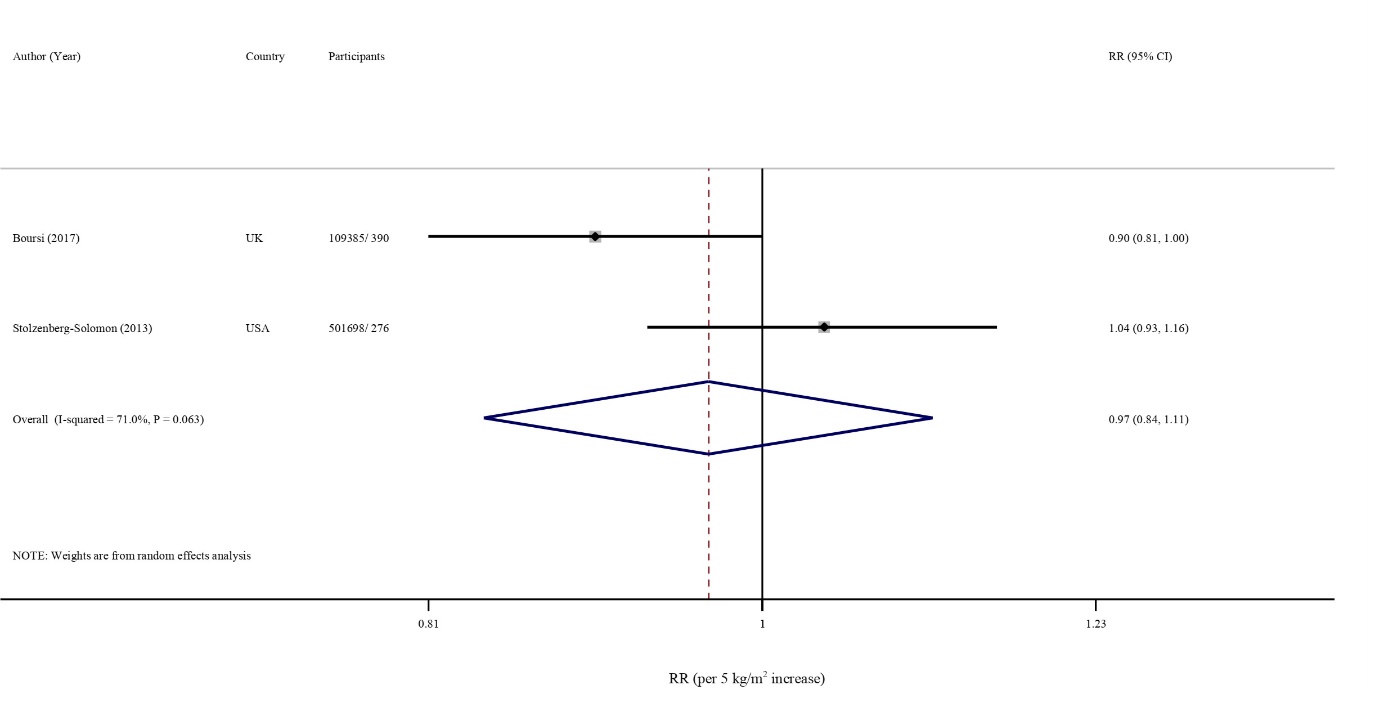


**Supplementary Figure S2**. Risk of pancreatic cancer associated with each 5-unit increase in body mass index in patients with T2D. The study-specific relative risk and 95 % CI are represented by the black square and horizontal line, respectively; the area of the black square is proportional to the specific-study weight to the overall meta-analysis. The center of the open diamond presents the pooled RR and its width represents the pooled 95 % CI. Weights are from the random-effects analysis


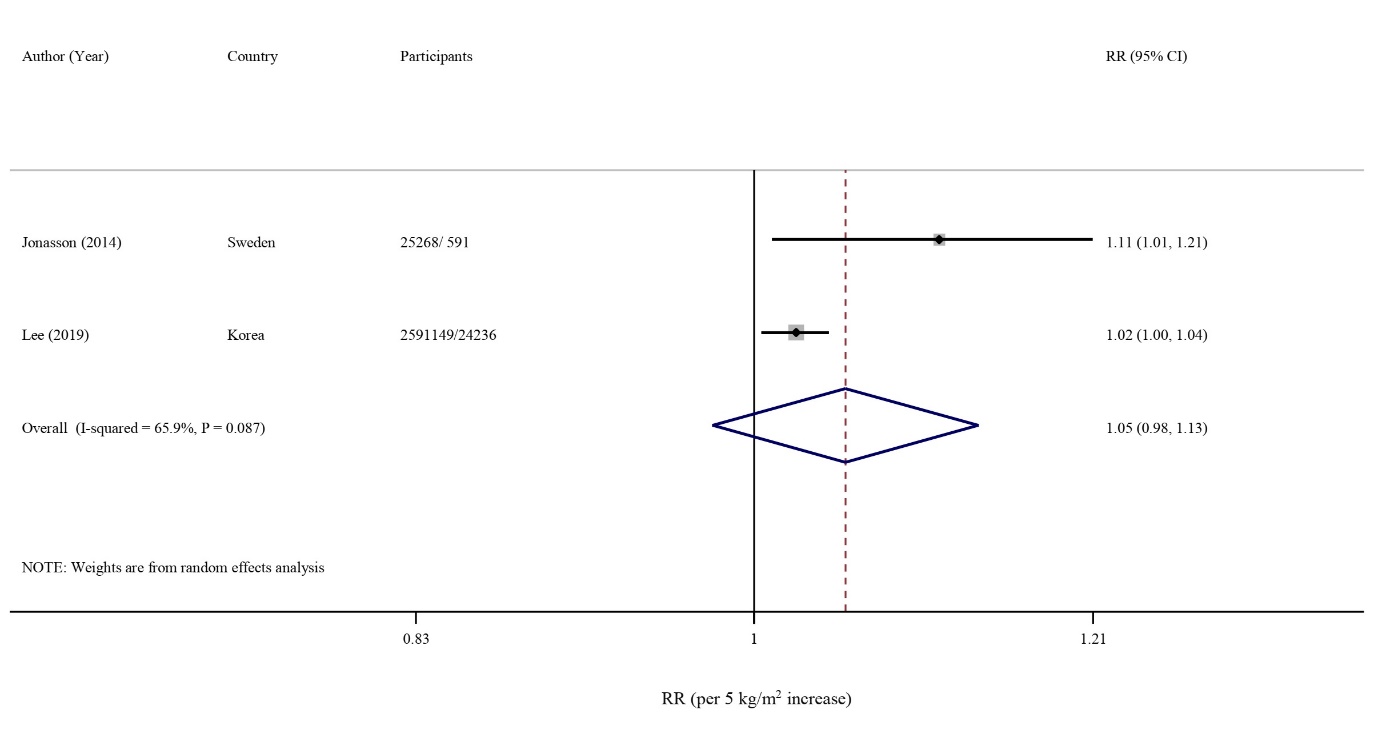


**Supplementary Figure S3**. Risk of colorectal cancer associated with each 5-unit increase in body mass index in patients with T2D. The study-specific relative risk and 95 % CI are represented by the black square and horizontal line, respectively; the area of the black square is proportional to the specific-study weight to the overall meta-analysis. The center of the open diamond presents the pooled RR and its width represents the pooled 95 % CI. Weights are from the random-effects analysis

**
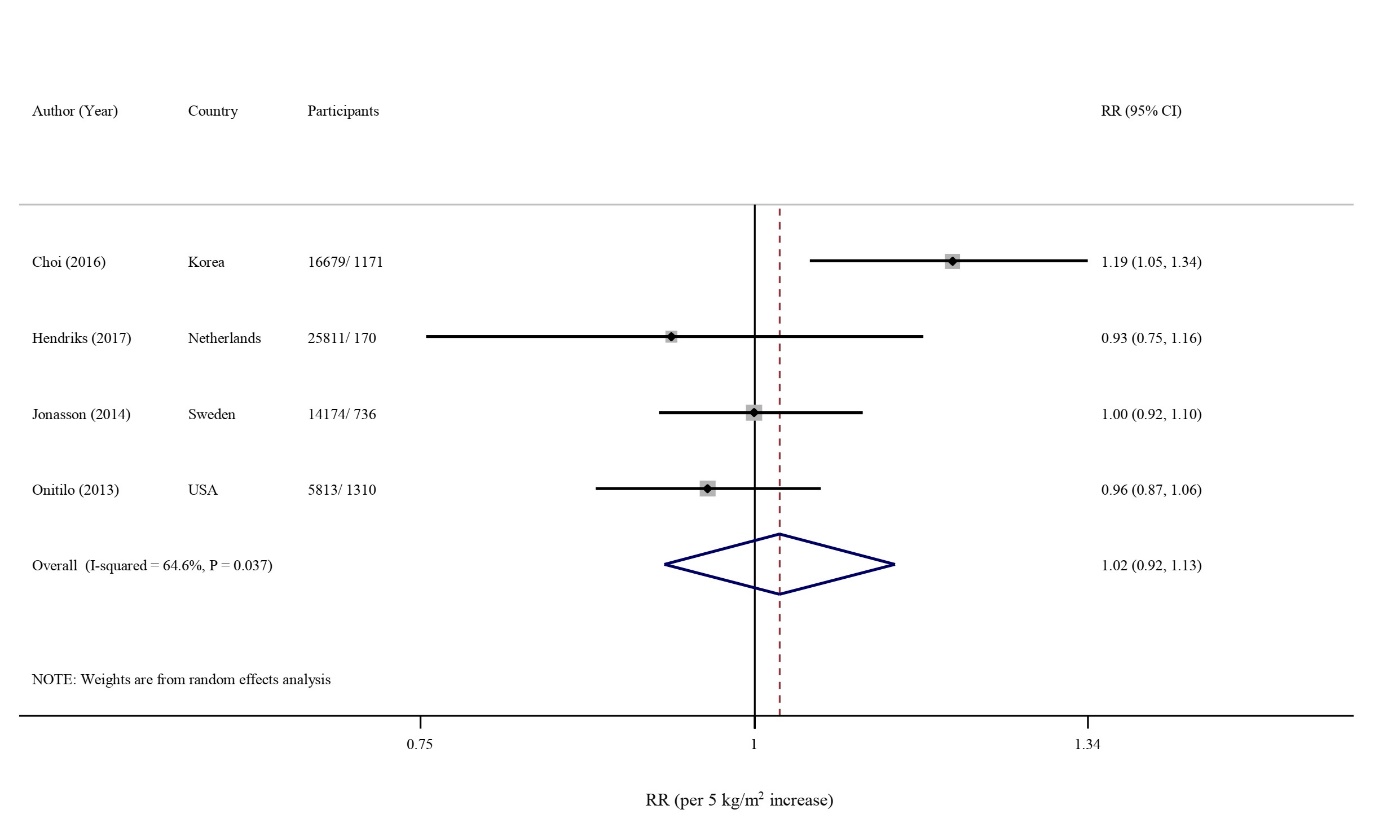
**

**Supplementary Figure S4.** Risk of prostate cancer associated with each 5-unit increase in body mass index in patients with T2D. The study-specific relative risk and 95 % CI are represented by the black square and horizontal line, respectively; the area of the black square is proportional to the specific-study weight to the overall meta-analysis. The center of the open diamond presents the pooled RR and its width represents the pooled 95 % CI. Weights are from the random-effects analysis


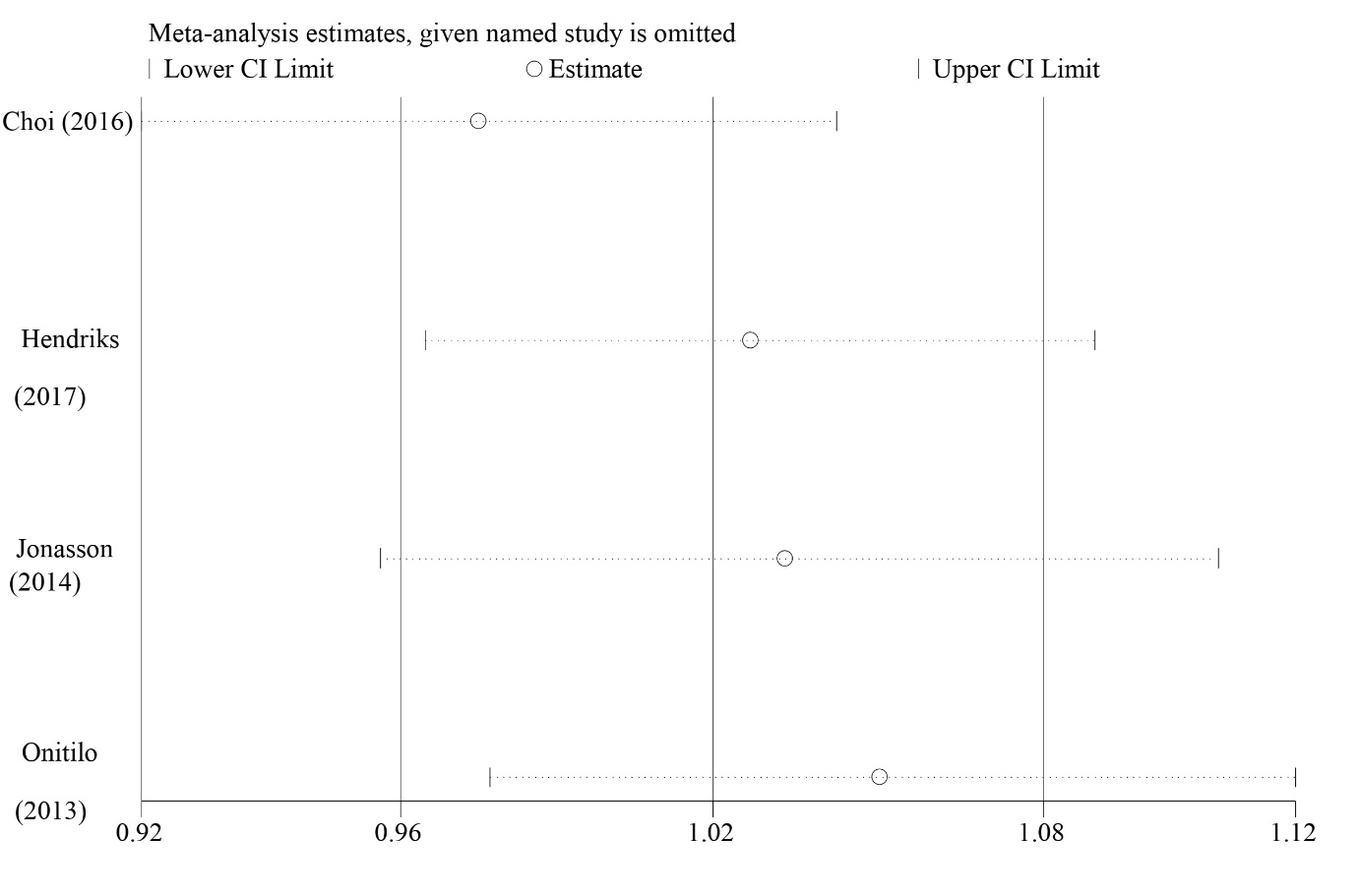


**Supplementary Figure S5.** Sensitivity analysis using the fixed-effects model for risk of prostate cancer in patients with T2D


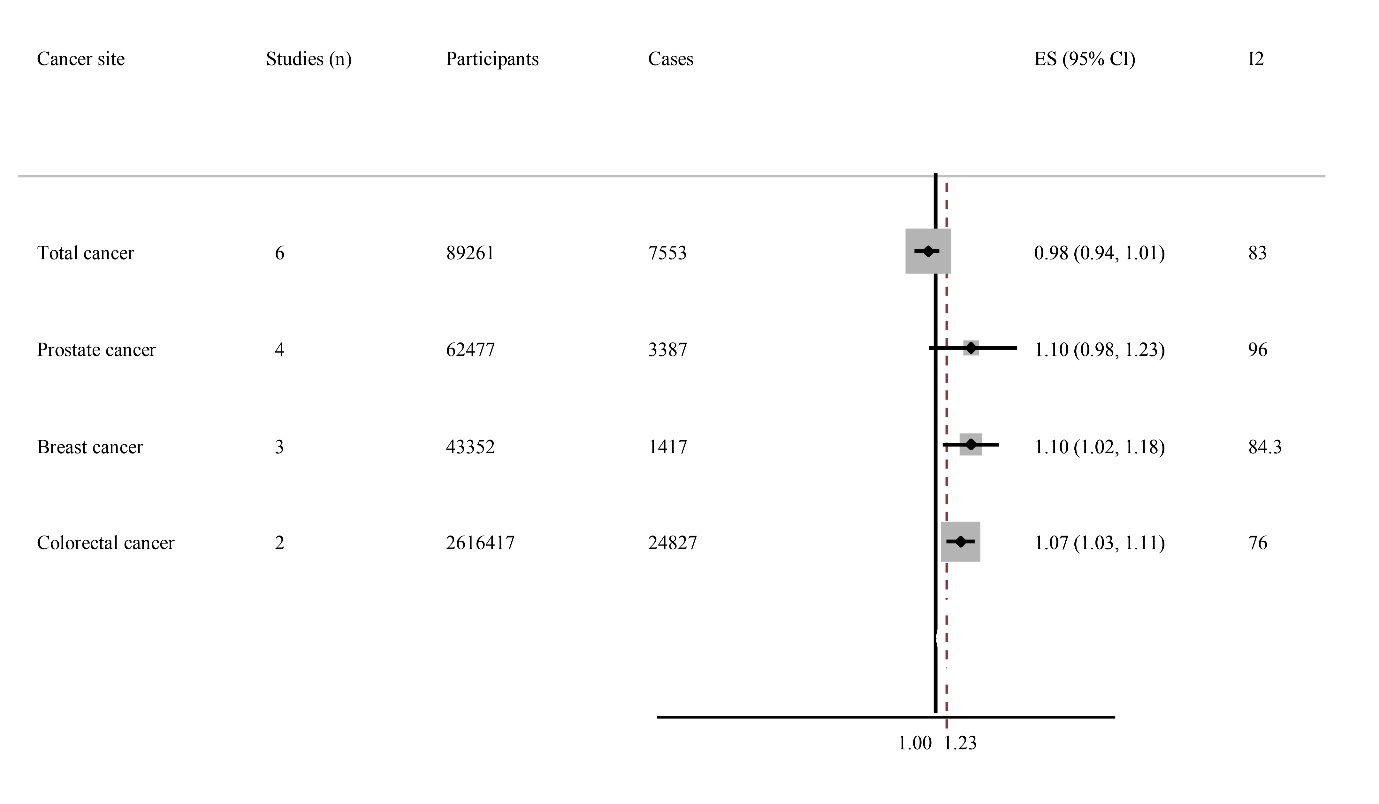


**Supplementary Figure S6.** Summary crude/unadjusted risk estimates by overall and cancer sites in per 5-unit increase in body mass index in patients with T2D. ES, effect size.
